# Comparing the Cost-Effectiveness of Campaigns Delivered *via* Various Combinations of Television and Online Media

**DOI:** 10.3389/fpubh.2018.00083

**Published:** 2018-03-23

**Authors:** Vanessa Allom, Michelle Jongenelis, Terry Slevin, Stacey Keightley, Fiona Phillips, Sarah Beasley, Simone Pettigrew

**Affiliations:** ^1^School of Psychology, Curtin University, Perth, WA, Australia; ^2^Education and Research, Cancer Council Western Australia, Perth, WA, Australia

**Keywords:** smoking cessation, cost-effectiveness, mass media, public health, digital media, television

## Abstract

**Background:**

Reflecting the increasing prevalence of online media, many mass media health campaigns are now delivered using both television (TV) and online media formats. The aim of this study was to evaluate a smoking cessation mass media campaign according to the cost-effectiveness of the various combinations of TV and online media formats to inform future media buying decisions.

**Methods:**

A quasi-experimental interrupted time series approach was employed. The campaign was delivered in seven 1-week bursts using TV, online video (OV), or online display (OD) (e.g., banner ads) formats in isolation and in various combinations over a 13-week period. Campaign bursts were separated by “off-weeks” in which no campaign materials were delivered. Assessed outcomes were the number of campaign response “events” recorded (campaign web page views, calls to a smoking cessation telephone service, and registrations for smoking cessation services). The cost-effectiveness of each individual and combined media format condition in terms of these outcome variables was calculated using attributed production and broadcasting costs.

**Results:**

Overall, OD alone was found to be the most cost-effective means of achieving the nominated campaign outcomes, followed by a combination of OV and OD and a combination of TV and OV. The use of TV in isolation was the least cost-effective.

**Conclusion:**

The results of this evaluation indicate that online media constitute a promising means of enhancing the cost-effectiveness of smoking cessation campaigns. Future research assessing a broader range of outcomes, especially smoking cessation, is needed to provide a more comprehensive account of the cost-effectiveness of various campaign media.

## Introduction

Reductions in smoking prevalence in many countries around the world have been attributed to the introduction of strict tobacco control policies and an array of public education strategies (1). As one method of education, mass media campaigns informing the public of the dangers of smoking have been recognized as an important contributor to these substantially reduced smoking rates (2–5). However, despite an overall reduction in smoking prevalence in high income countries, the burden of the disease associated with tobacco use remains high, with approximately 15% of health care expenditure attributed to smoking (6). There is therefore a need to continue to develop and implement effective mass media campaigns to further reduce tobacco use.

As funding for mass media smoking cessation campaigns is generally limited, the cost-effectiveness of program delivery is an important consideration. One of the factors that influence cost-effectiveness is the media format used to deliver the campaign. Traditional formats utilized in mass media campaigns include broadcast television (TV), radio, print, and outdoor advertising (2–4, 7). A review of mass media smoking cessation campaigns using these formats concluded that such campaigns can successfully encourage quitting and reduce the prevalence of smoking (2). Broadcast TV appears to be an especially effective format, with evidence suggesting that campaigns are more likely to be recalled and to generate calls to a smoking cessation service when delivered *via* broadcast TV compared with radio (8–10). However, TV campaigns are costly (7), which limits the cost-effectiveness of TV as a medium. Newer online media formats have evolved that are relatively inexpensive (11, 12), potentially offering complementary means of delivering mass media smoking cessation campaigns.

Audiences are increasingly engaging with online media in addition to or as an alternative to broadcast TV (13, 14). Correspondingly, online platforms are now commonly used for health promotion (15), and many major public health organizations are using social media sites to communicate health messages (12, 16–18). In addition to being relatively inexpensive, online media may offer a suitable platform for targeting population segments that exhibit higher smoking rates (19, 20). For example, lesbian, gay, and bisexual people tend to have higher smoking rates and they have been found to use online media more than TV (21). In addition, young people are an important group to target because of the particular need to discourage initiation at this stage of the lifecycle (22, 23) and because they remain a substantial proportion of the smoking population (24). Members of this age group engage less with live broadcast TV (25) and spend a greater number of hours online in a typical week than older people (26). Mass media campaigns that utilize online platforms may therefore have the potential to reach target groups that may be less likely to frequently engage with TV. They also have the potential to encourage greater audience interaction with related online content (27). A further consideration is that individuals are exposed to online tobacco advertising, which has been found to influence their attitudes, intentions, and behaviors (28–30), and as such the online environment constitutes an appropriate environment for tobacco control messaging. However, some population groups have limited online access or disabilities that impair access to online media, and therefore campaigns delivered exclusively *via* online media may not adequately reach vulnerable groups (31, 32).

Given the advantages and disadvantages associated with individual media formats, delivering campaigns *via* combinations of media may optimize campaign cost-effectiveness (33). Indeed, mass media smoking cessation campaigns delivered using multiple media formats including TV, radio, print, and online have been found to be cost-effective (7, 34, 35). However, few evaluations of smoking cessation campaigns that utilize multiple media formats for message dissemination have attempted to systematically compare outcomes according to whether campaigns are delivered *via* multiple or individual media (17, 36). One study demonstrated that higher online dosage of a campaign led to greater awareness of the online ad, while higher TV dosage resulted in greater awareness of both the online and televised ads (37). The authors concluded that while online video (OV) was a more cost-effective means of generating awareness, TV produced greater awareness overall and thus OV should be used as an adjunct to TV to optimize outcomes. Another study found that using print, radio, and online together was more cost-effective than using print or radio in isolation but less cost-effective than using online in isolation, suggesting that online media alone may be the most cost-effective approach (38). While informative, this analysis did not include TV, which is an important omission because TV remains a major source of entertainment and information (39).

Given the varying costs associated with disseminating campaign messages *via* different media, an evaluation of the relative and combined cost-effectiveness of online and TV formats is needed to provide insight into methods of maximizing limited campaign funds. Therefore, the aim of this study was to evaluate the cost-effectiveness of delivering a mass media smoking cessation campaign using TV and two forms of online media in isolation and in combination to test the independent and synergistic effects of these formats.

## Methods

### Design

A quasi-experimental evaluation design was adopted in which a mass media smoking cessation campaign was delivered in Western Australia from April to July 2015 using three media formats across seven conditions. The population of Western Australia in 2015 was approximately 2.6 million, 13% of whom were daily smokers (40). The specific media formats included in the study were TV, OV, and online display (OD) in varying combinations. In three of the seven conditions, the campaign was delivered by each of the three media in isolation: TV alone, OV alone, and OD alone. Three further conditions used all possible combinations of two formats (TV + OV, TV + OD, and OV + OD). In the final condition, all three formats (TV + OV + OD) were used to deliver the campaign. The order of conditions was determined by random allocation (see Table 1 for media activity by campaign week).

**Table 1:** Media activity by campaign week.

Media activity	Campaign week																
	1	2	3	4	5	6	7	8	9	10	11	12	13	14	15	16	17
Television			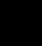		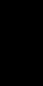						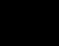		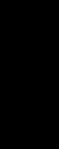				
Online video									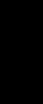						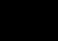		
Online display							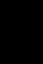				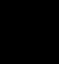						

*Black cells represent media activity*.

For conditions that included the campaign in TV format, two 30-s advertisements and three 15-s advertisements were broadcast on Western Australian metropolitan and regional TV stations. For conditions that included the campaign in OV format, the same five advertisements were played on various news and entertainment websites (e.g., YouTube, Facebook, and TV program streaming websites) and mobile application (app) versions of these sites. These OVs were played automatically before or during OV content and in the ad breaks of online TV programming (i.e., “catch-up TV”). For conditions including online display (OD) as a format, campaign materials were shown as online static and animated ad placements appearing within web pages or news feeds. OD ads were placed on the same websites and mobile apps as the OV ads.

To prevent the potential for campaign novelty to influence the results of the earlier bursts relative to the later bursts, the same campaign was aired earlier in the year from February to March 2015. The standard evaluation of this prior campaign wave indicated that it was effective in terms of attracting the target audience’s attention and encouraging quitting-related thoughts and behaviors, and was generally considered believable and personally relevant by smokers (unpublished data). As such, this campaign represented an appropriate tool for subsequently assessing the relative cost-effectiveness of various campaign media used to disseminate the campaign.

Campaign bursts during the testing period were separated by “off-weeks” in which no campaign materials were delivered to provide a wash-out period. Campaign response events (described in detail below) were collected on a weekly basis, commencing the 2 weeks preceding the campaign period and ending 2 weeks after the campaign concluded. The pre-campaign data formed the comparison point for results occurring during the “on weeks.” It was not possible to include a no-exposure control group as other smoking cessation campaigns were being implemented in other Australian states at the time, preventing these locations from constituting potential comparison sites.

### Campaign Materials

For the purposes of this experiment, the “16 Cancers” campaign was delivered in Western Australia as part of Cancer Council Western Australia’s *Make Smoking History* initiative. The campaign content highlights that smoking is a causal factor in the development of 16 specific types of cancer and that quitting smoking can reduce risk.^1^ Emotionally intense messages and graphic imagery show people experiencing the effects of smoking-related cancers across five scenes. The scenes included a man being diagnosed with lung cancer, a man having surgery for bowel cancer, a woman with a urostomy “bag” due to bladder cancer, a man experiencing difficulty talking due to throat cancer, and a woman eating *via* a tube due to stomach cancer. The two 30-s ads included three of the scenes while the three 15-s ads each featured one scene. At the end of each ad, contact details are provided for a smoking cessation telephone counseling service (Quitline) and the *Make Smoking History* website.^2^ The website provides information on quitting smoking and access to other smoking cessation services including an online quit tool (QuitCoach^3^) and a mailed out smoking cessation resource (Quit Kit).

Routine campaign evaluation research undertaken when “16 Cancers” was aired previously in Western Australia found that three-quarters of smokers who recalled seeing the ad reported that it was convincing (77%) and made them seriously consider quitting (74%: unpublished data). These favorable results are likely to translate into campaign effectiveness given previous research demonstrating the strong relationship between perceived effectiveness of smoking cessation advertisements and actual cessation attempts (41, 42).

### Costs

Costs of the campaign included those associated with producing and broadcasting the campaign materials. These costs were summed for each burst to compute total cost per media condition. The production costs were apportioned across the media formats according to the costs that would be incurred should each condition occur separately. For example, TV and OV used largely the same video materials, so these costs were distributed differently according to condition. Where these two media were used individually, they each attracted the total production cost to reflect the single-media condition. Where the two media were used in combination, the production costs were divided equally between them.

### Effectiveness

Effectiveness was measured by the number of campaign response “events” attributed to each media format. These included the following: (1) unique page views of the “16 cancers” campaign page on the *Make Smoking History* website, (2) calls to the Quitline, (3) online registrations for QuitCoach, and (4) requests for a Quit Kit (which could occur *via* telephone or the website).

### Campaign Awareness

In addition, campaign awareness was measured using a telephone survey that was administered to independent samples of approximately 100 respondents during the off-weeks after each burst. Computer assisted telephone interviewing was undertaken using random digit dialing. Quotas were established for each round of data collection to generate a sample with equal representation of smokers and non-smokers, males and females, and those in the following age categories: 25–34, 35–45, and 45–55 years (25–55 years was the stated target group for the campaign). Non-smokers were included in the survey sample to account for the possibility that non-smokers were contributing to effectiveness data (i.e., non-smokers may have accessed the campaign website to source material for friends or family members). A location quota was also applied to achieve a sample comprising 70% metropolitan and 30% regional residents to reflect the distribution of the Western Australian population. Campaign awareness was calculated as the percentage of individuals surveyed who recalled or recognized the campaign. Given the small survey sample size for each condition (*n* = 100), it was not possible to assess actual quitting rates in response to campaign exposure. The study was approved by a University Human Research Ethics Committee.

### Analyses

Cost-effectiveness of each condition was operationalized as cost per weighted total additional events. Additional events were calculated as the number of events recorded during each campaign burst minus the number of events recorded at baseline (2 weeks before the first campaign burst). As per Clayforth et al. (38) additional events were weighted to reflect the level of effort required to make these actions. Making a call to the Quitline, registering for QuitCoach, or requesting a Quit Kit involves communicating with health professionals or signing up to a cessation service. As these actions are more effortful and potentially more likely to result in a quit attempt than visiting a web page, they were given greater weight in the present analyses than web site views. Protocols used in previous research (38) were followed in the current analysis to determine weighting as there exist limited data on conversion of website engagement into quitting-related behavior upon which to base assumptions (12). Weightings were applied using the following procedure: the numbers of Quitline calls, QuitCoach registrations, and Quit Kit requests recorded over the entire campaign period were summed and divided by the number of unique page views recorded over the entire campaign period. Additional unique page views for each condition were then multiplied by this number to achieve weighted page views (38). The formula for weighted additional unique page views (*y*) was therefore:

y=x*[(a+b+c)/d] ,

where *x* is the additional unique page views per condition, *a* is the campaign total Quitline calls, *b* is the campaign total QuitCoach registrations, *c* is the campaign total Quit Kit requests, and *d* is the campaign total unique page views. The weighted total (*z*) was then calculated according to the following formula:

z=y+e+f+g,

where *y* is the weighted additional page views per condition, *e* is the additional Quitline calls per condition, *f* is the additional QuitCoach registrations per condition, and *g* is the additional Quit Kit requests per condition.

Sensitivity analyses with varying weighting assumptions were conducted to determine the robustness of the cost-effectiveness calculation using the following different weighting assumptions: (1) half the original formula used to weight unique page views (giving less weight to page views), (2) double the original formula used to weight unique page views (giving more weight to page views), (3) weighting the unique page views *and* calls to the Quitline by QuitCoach registrations and Quit Kit requests instead of only weighting unique page views, (4) using all page views instead of only unique page views, (5) using the off-week before each campaign burst as the baseline instead of the 2 weeks before the first campaign burst as the baseline, and (6) dividing the weighted total additional events for each condition by the awareness of the campaign for that condition. Further, incremental cost-effectiveness ratios (43) were calculated for every possible comparison between media formats and combinations.

## Results

### Costs and Events

The costs, rates of awareness, and events recorded over the campaign period are displayed in Table 2. OD was the least expensive condition and TV + OV + OD was the most expensive. Across the various conditions, the numbers of unique page views were substantially higher in weeks the campaign was delivered *via* an online format than when delivered *via* TV, with the OV + OD condition resulting in the most unique page views and the TV condition the least. Reflecting the extra effort required to call the Quitline (i.e., it was not possible to click through from an online ad) and the provision of Quitline information on all cigarette packs (i.e., Quitline calls could have been generated from non-campaign materials), the gaps between on- and off-weeks were less apparent for Quitline events. However, when the Quitline data were examined by either only on-week results or combined on-week and following off-week results, OD resulted in the greatest number of Quitline calls and OV + OD the least. For QuitCoach registrations and Quit Kit requests, the numbers of events were relatively small and no clear patterns emerged. The highest level of campaign awareness among survey respondents was recorded for the TV + OV condition and the lowest was for OD.

**Table 2:** Campaign costs, awareness, and events by media condition.

Media condition	Costs (AUD)			Awareness (%)	Events (*n*)			
	Production	Broad casting	Total		Unique page views	Quitline calls	QuitCoach registrations	Quit Kit requests
Baseline					69	54	5	2
Off-week					79	57	5	4
TV	184,493	68,496	252,989	66	139	69	10	9
Off-week					232	56	4	8
TV + OV	188,970	110,426	299,397	77	8,774	72	7	10
Off-week					383	55	9	4
OD	1,932	26,361	28,293	41	4,635	89	4	10
Off-week					714	74	12	11
OV + OD	187,622	68,291	255,914	67	12,284	45	11	11
Off-week					692	57	8	10
TV + OD	186,425	94,857	281,282	60	5,716	68	11	5
Off-week					778	66	14	11
TV + OV + OD	190,902	136,787	327,689	73	8,434	78	10	13
Off-week					706	74	9	14
OV	185,690	41,930	227,621	61	6,836	58	2	5
Off-week					201	77	4	7
Off-week					95	49	8	8
Average	160,862	78,164	239,026	64				

Total					50,767	1,098	133	142

*TV, television; OV, online video; OD, online display; AUD, Australian dollars*.

### Additional Events

Additional events for each media condition, raw and weighted, are displayed in Table 3. The OV + OD condition resulted in the greatest number of additional events, which was primarily driven by unique page views. The TV condition resulted in the fewest number of additional events. Recalculating total additional events with campaign page views weighted, OV + OD still produced the greatest number of additional events, followed by TV + OV + OD and then TV + OV.

**Table 3:** Additional events recorded for each media condition.

Media condition	Additional events					Weighted total events
	Unique page views (weighted)	Quitline calls	QuitCoach registrations	Quit Kit requests	Total events	
OV + OD	12,215 (330)	−9	9	6	12,221	336
TV + OV + OD	8,365 (226)	24	11	5	8,405	266
TV + OV	8,705 (235)	18	8	2	8,733	263
OV	6,767 (183)	4	3	−3	6,771	187
TV + OD	5,647 (153)	14	3	6	5,670	176
OD	4,566 (123)	35	8	−1	4,608	165
TV	70 (2)	15	7	5	97	29

*TV, television; OV, online video; OD, online display; additional events, events in each campaign burst—baseline; weighted total, weighted unique page views + Quitline calls + QuitCoach registrations + Quit Kit requests*.

*Conditions are listed in order of additional events achieved*.

### Cost-Effectiveness

Table 4 displays the cost-effectiveness of each condition. All amounts are specified in Australian dollars. Cost-effectiveness data were not calculated for individual outcomes that did not achieve more events than baseline. Overall, OD was the most cost-effective condition ($171 per additional weighted event) and TV the least ($8,724). Cost-effectiveness for TV improved when it was combined with other media formats (TV + OV: $1,137, TV + OV + OD: $1,231, TV + OD: $1,598).

**Table 4:** Cost-effectiveness of each media condition ($AUD).

Media condition	Cost/additional events				Cost/weighted total
	Cost/unique page view (weighted)	Cost/Quitline call	Cost/QuitCoach registration	Cost/Quit Kit request	
OD	6 (230)	808	3,537	n/a	171
OV + OD	21 (775)	n/a	28,435	42,652	762
TV + OV	34 (1,274)	16,633	37,425	149,698	1,138
OV	34 (1,244)	56,905	75,874	n/a	1,217
TV + OV + OD	39 (1,450)	13,654	29,790	65,538	1,232
TV + OD	50 (1,838)	20,092	93,761	46,880	1,598
TV	3,614 (126,495)	16,866	36,141	50,598	8,724

*TV, television; OV, online video; OD, online display; weighted total, weighted unique page views + Quitline calls + QuitCoach registrations + Quit Kit requests; n/a, could not be calculated due to fewer events than baseline recorded*.

*All events are additional events; all values are in Australian dollars (AUD); conditions are listed in order of cost-effectiveness*.

When examining the cost of specific types of events, there were varying trends by condition. In terms of weighted unique page views, TV was the least cost-effective at $126,495 per page view and OD was the most cost-effective at $230 per page view. Any condition that included TV resulted in a greater cost per unique page view compared with other conditions that did not include TV. Regarding calls to the Quitline, OD was the most cost-effective condition ($808 per call) and OV the least ($56,905 per call). For Quit Kit requests, the OV + OD condition was the most cost-effective means of encouraging engagement with this service ($42,652 per request), but the conditions in which these formats were used alone did not result in additional Quit Kit requests.

### Sensitivity Analysis Results

Overall, the results were robust for alternative weighting assumptions and when accounting for level of campaign awareness. Specific differences by sensitivity analysis type were relatively minor (see Table 5). The TV + OV + OD condition became more cost-effective than the OV condition in sensitivity analyses 1 (half the original formula used to weight unique page views: $2,140 vs $2,383), 3 (weighting the unique page views *and* calls to the Quitline by QuitCoach registrations and Quit Kit requests: $4,867 vs $6,044), and 5 (using the off-week before each campaign burst as the baseline: $1,510 vs $1,701). Analysis 2 (double the original formula used to weight unique page views) resulted in reduced cost per additional event compared with the original analysis, but yielded the same pattern of results in terms of the relative cost-effectiveness of each condition. Analysis 4 (using all page views instead of only unique page views) resulted in OV becoming more cost-effective than TV + OV ($928 vs $954). In analysis 6 (dividing the weighted total additional events for each condition by the awareness of the campaign for that condition), OV became relatively less cost-effective compared with TV + OV + OD ($1,995 vs $1,686).

**Table 5:** Cost-effectiveness of each media condition: original analysis and sensitivity analyses.

Media condition	Original analysis	Sensitivity analysis					
		1	2	3	4	5	6
OD	171	273	98	699	156	189	417
OV + OD	761	1,495	384	3,243	646	905	1,136
TV + OV	1,137	2,055	600	4,855	954	1,188	1,476
OV	1,217	2,383	615	6,044	928	1,701	1,995
TV + OV + OD	1,231	2,140	665	4,867	1,042	1,510	1,686
TV + OD	1,601	2,831	856	6,527	1,423	1,942	2,668
TV	8,756	9,053	8,218	15,679	8,740	10,710	13,267

*TV, television; OV, online video; OD, online display; 1, half formula used to weight page views; 2, double formula used to weight page views; 3, unique page views and Quitline calls weighted instead of only unique page views; 4, all page views instead of only unique page views; 5, using “off-week” before each burst as the baseline instead of 2 weeks before first campaign burst as the baseline; 6, dividing weighted additional events by awareness*.

*All values are in Australian dollars; conditions are listed in order of cost-effectiveness by original analysis*.

**Figure 1:**
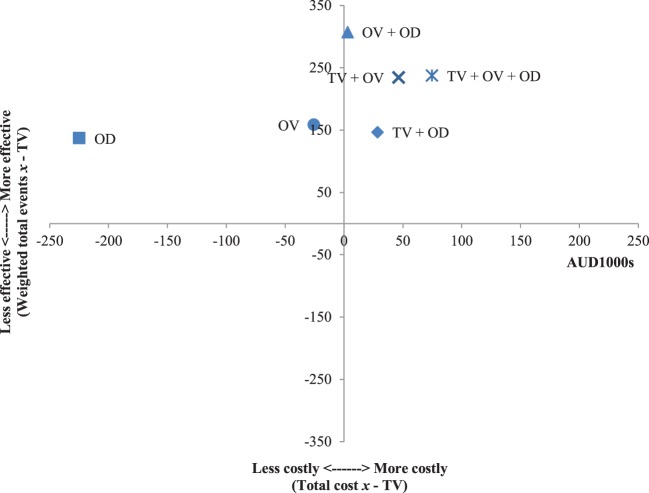
Incremental cost-effectiveness of each format compared with television (TV). The cost-effectiveness of TV forms the baseline (cross-section of *x* and *y* axes). Effectiveness is weighted total events of each format (*x*) compared with TV. Cost is total costs in AUD1,000s of each format (*x*) compared with TV. Data points that fall in the upper left quadrant represent formats that are more cost-effective than TV.

**Figure 2:**
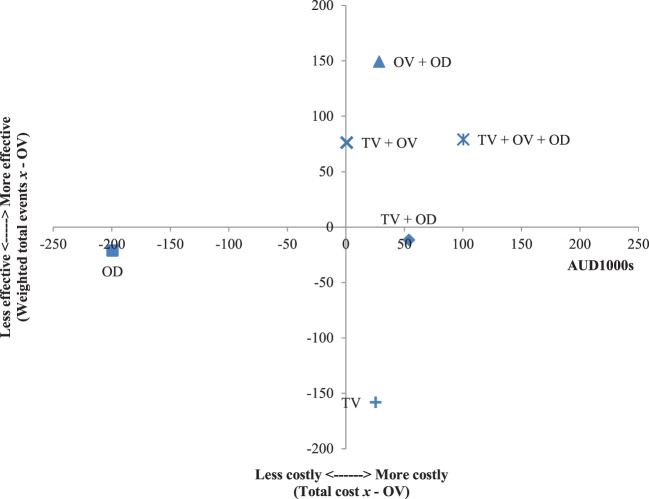
Incremental cost-effectiveness of each format compared with online video (OV). The cost-effectiveness of OV forms the baseline (cross-section of *x* and *y* axes). Effectiveness is weighted total events of each format (*x*) compared with OV. Cost is total costs in AUD1,000s of each format (*x*) compared with OV. Data points that fall in the upper left quadrant represent formats that are more cost-effective than OV.

**Figure 3:**
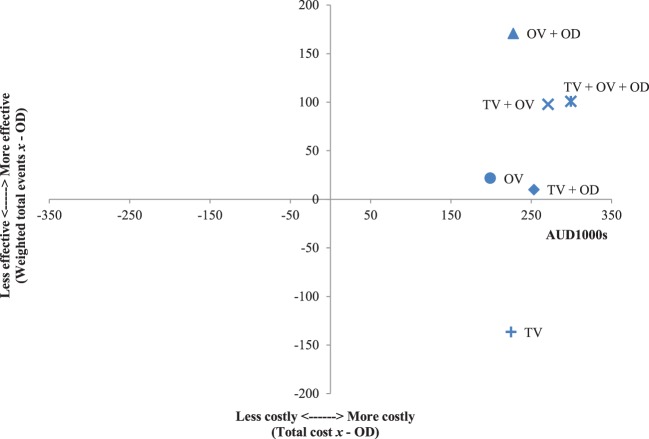
Incremental cost-effectiveness of each format compared with online display (OD). The cost-effectiveness of OD forms the baseline (cross-section of *x* and *y* axes). Effectiveness is weighted total events of each format (*x*) compared with OD. Cost is total costs in AUD1,000s of each format (*x*) compared with OD. Data points that fall in the upper left quadrant represent formats that are more cost-effective than OD.

**Figure 4:**
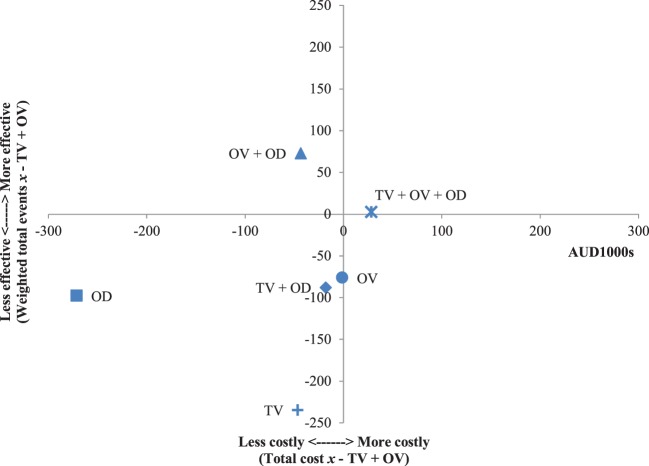
Incremental cost-effectiveness of each format compared with television (TV) + online video (OV). The cost-effectiveness of TV + OV forms the baseline (cross-section of *x* and *y* axes). Effectiveness is weighted total events of each format (*x*) compared with TV + OV. Cost is total costs in AUD1,000s of each format (*x*) compared with TV + OV. Data points that fall in the upper left quadrant represent formats that are more cost-effective than TV + OV.

**Figure 5:**
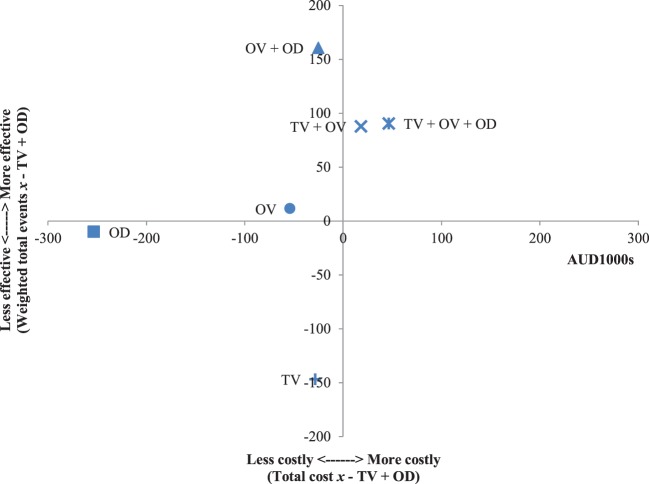
Incremental cost-effectiveness of each format compared with television (TV) + online display (OD). The cost-effectiveness of TV + OD forms the baseline (cross-section of *x* and *y* axes). Effectiveness is weighted total events of each format (*x*) compared with TV + OD. Cost is total costs in AUD1,000s of each format (*x*) compared with TV + OD. Data points that fall in the upper left quadrant represent formats that are more cost-effective than TV + OD.

**Figure 6:**
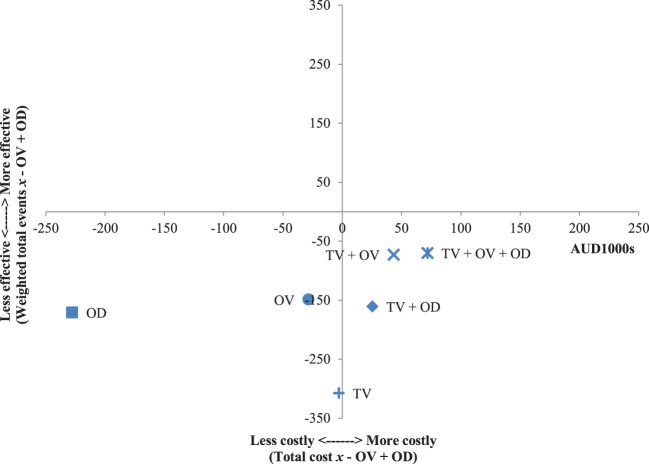
Incremental cost-effectiveness of each format compared with online display (OD) + online video (OV). The cost-effectiveness of OV + OD forms the baseline (cross-section of *x* and *y* axes). Effectiveness is weighted total events of each format (*x*) compared with OV + OD. Cost is total costs in AUD1,000s of each format (*x*) compared with OV + OD. Data points that fall in the upper left quadrant represent formats that are more cost-effective than OV + OD.

**Figure 7:**
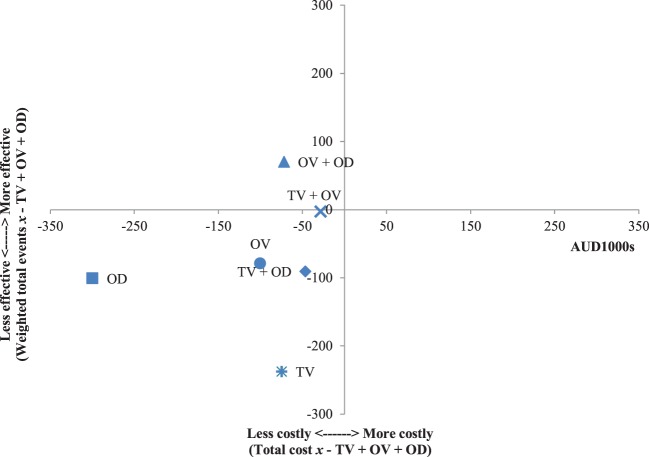
Incremental cost-effectiveness of each format compared with television (TV) + online display (OD) + online video (OV). The cost-effectiveness of TV + OV + OD forms the baseline (cross-section of *x* and *y* axes). Effectiveness is weighted total events of each format (*x*) compared with TV + OV + OD. Cost is total costs in AUD1,000s of each format (*x*) compared with TV + OV + OD. Data points that fall in the upper left quadrant represent formats that are more cost-effective than TV + OV + OD.

The results of the incremental cost-effectiveness ratios calculated for every possible combination of media formats are shown in Figures 1–7. These analyses provide a graphical representation of the average incremental cost that is associated with an additional unit of the measured effect for each study condition. The figures can be interpreted as follows: (1) any data point that falls in the top two quadrants indicates that the corresponding media format is more effective than the media format used as baseline and (2) any data point that falls in the left two quadrants indicates that the corresponding media format is less expensive than the media format used as baseline. Therefore, any data point that falls in the top left quadrant indicates a more cost-effective media format than the media format used as baseline, and correspondingly, any data point that falls in the bottom right quadrant indicates a less cost-effective media format(s) than the media format used as baseline. The primary results illustrated by the figures are as follows. Figure 1 shows that OD alone and OV alone were both more cost-effective formats than TV alone. Figure 4 shows that the OV + OD condition was more cost-effective than the TV + OV condition. Figure 5 illustrates that OV alone and OV + OD were more cost-effective combinations than TV + OD, while Figure 7 shows that OV + OD was more cost-effective than TV + OD + OV.

## Discussion

The costs of health promotion campaigns are largely determined by decisions relating to the media platforms that will be used to disseminate the messages. As such, media cost-effectiveness is a critical issue. This study assessed the relative cost-effectiveness of delivering a mass media smoking cessation campaign using various combinations of media formats including TV, OV, and OD. Overall, OD when used alone was found to be the most cost-effective means, relative to the other included formats, by which to generate a specific combination of campaign outcomes (i.e., website campaign page views, calls to a smoking cessation telephone service, downloads of a smoking cessation package, and registrations for a smoking cessation service). The next most cost-effective conditions were OV + OD and TV + OV. TV alone was the least cost-effective condition in terms of the particular outcome measures assessed. Using alternative weighting assumptions and accounting for level of campaign awareness yielded generally consistent results, suggesting the findings are reliable.

The finding that OD alone was relatively more cost-effective than other conditions is similar to Clayforth et al.’s results (38) where delivering a smoking cessation campaign *via* online materials only was more cost-effective than using online, radio, and print formats in combination and radio and print in isolation. This outcome is encouraging for organizations that face financial barriers to delivering mass media campaigns using TV or OV (2, 44). However, TV is likely to remain an important media format for future tobacco control campaigns as it has played a key role in effective tobacco control campaigns to date (2, 3), and it remains a major source of entertainment and information for many people (e.g., 86% of Australians in the first quarter of 2016 watched some broadcast TV including free-to-air and/or subscription channels on in-home TV sets each week) (39). Further, TV has the ability to reach members of disadvantaged groups who may not have access to online media (32), and TV advertising is likely to have other important benefits such as softening the ground for more stringent tobacco control policies (45, 46). Adding online formats to TV resulted in much improved cost-effectiveness, reflecting the relatively low cost of creating an OV or display ad from a TV ad but the large gains achieved from using multiple formats over TV alone. The results of this study thus highlight the benefits to be gained from complementing TV ads with online materials.

### Strengths and Limitations

The primary strength of this study was the ability to separate independent and synergistic effects to provide guidance to campaign managers when scheduling advertisements across different media. However, there are several limitations to consider when interpreting the results. First, the order of the seven study conditions may have resulted in greater effects being recorded in the latter bursts of the campaign as a result of additive effects that were not present in the first burst. However, the study design ensured that additive effects were minimized given that the same campaign had been delivered very recently using the same media formats. The variations in effects were not linear indicating that this approach was successful.

Similarly, as the campaign was delivered across time rather than in one burst simultaneously to multiple groups, it cannot be determined whether events recorded during each burst were exclusively due to the current campaign activity. Previous research demonstrated that a higher dosage of a campaign *via* TV led to increased awareness of both TV and online material, while a higher dosage of a campaign *via* online media only increased awareness of online material (37). Therefore, a partial explanation for the strong performance of OD in this study could be that exposure to the longer ads delivered *via* TV and OV increased the familiarity of the “16 Cancers” campaign, thereby increasing the likelihood of audiences choosing to click through to the campaign website when exposed to materials *via* OD. Future research could address this issue by including multiple groups of participants who are exposed to only one of the delivery formats or combinations of formats (e.g., the same campaign could be run in multiple states using different media schedules).

Third, effectiveness was operationalized using a select group of outcomes that may not have captured the full range of smoking cessation actions initiated by the campaign (e.g., visits to medical practitioners to discuss quitting options, seeking information relating to quitting from alternative sources, and reducing tobacco intake). In particular, future research would benefit from including actual smoking cessation as an outcome (47), which was not able to be assessed in this study.

Fourth, the correlation between format type and outcomes used to assess effectiveness may have influenced the relative cost-effectiveness of some formats. For example, online materials allow immediate access to the campaign website through a click, while TV is a step removed. Although comprehensive sensitivity analyses were conducted and weighting was used to balance the contribution of website views with more effortful outcomes, website views remained a dominant determinant of outcomes, which is likely to have disadvantaged TV by enhancing the overall effectiveness of conditions including an online delivery format. In addition, it is possible that the weighting assumptions employed do not accurately reflect the likelihood that campaign page views vs calls and registrations to smoking cessation services are translated into quitting-related behavior. Compared with page views, calls and registrations represent more downstream actions that may occur later in time and are likely to be more closely associated with cessation. An important area of future research is assessing the relationships between different cessation-related behaviors to inform more precise cost-effectiveness calculations. Finally, the cost data used were specific to the “16 Cancers” campaign and may not generalize to other campaigns that incur different production and distribution costs.

### Conclusion

The results of this study provide evidence of the independent and synergistic effects of delivering a mass media smoking cessation campaign using TV, OV, OD, and all combinations of these formats. OD alone was found to be the most cost-effective condition in terms of the specific outcomes measured in the study. While TV alone demonstrated relatively low cost-effectiveness, formats in which TV and OV were combined produced synergistic effects and represented more cost-effective options than when these media formats were used in isolation. The findings provide potential guidance for campaign managers in their efforts to employ cost-effective mass media smoking cessation campaigns.

## Data Availability Statement

The dataset is available upon request. Please contact Fiona Phillips: fphillips@cancerwa.asn.au.

## Ethics Statement

This study was approved by the Curtin University Human Research Ethics Committee. Individuals responding to the telephone survey provided verbal consent to participate in the study.

## Author Contributions

VA took primary responsibility for preparing the manuscript including performing the analyses and SP managed the study; SK, TS, FP, SP, and MJ conceptualized the study; FP and SB managed data collection; all the authors assisted with manuscript preparation and approved the final manuscript for submission.

## Conflict of Interest Statement

During the study VA, SK, TS, FP, and SB were employed by the Cancer Council Western Australia, and SP and MJ were employed by a research unit that is partly funded by the Cancer Council Western Australia.

## Footnotes

^1^https://makesmokinghistory.org.au/more-information/past-campaigns/16-cancers (accessed February 17, 2017).

^2^https://makesmokinghistory.org.au (accessed February 17, 2017).

^3^www.quitcoach.org.au (accessed February 17, 2017).
